# Calcium and Nuclear Signaling in Prostate Cancer

**DOI:** 10.3390/ijms19041237

**Published:** 2018-04-19

**Authors:** Ivan V. Maly, Wilma A. Hofmann

**Affiliations:** Department of Physiology and Biophysics, Jacobs School of Medicine and Biomedical Sciences, University at Buffalo, 955 Main Street, Buffalo, NY 14203, USA; ivanmaly@buffalo.edu

**Keywords:** metastasis, nuclear import, myosin IC, calcium, prostate cancer

## Abstract

Recently, there have been a number of developments in the fields of calcium and nuclear signaling that point to new avenues for a more effective diagnosis and treatment of prostate cancer. An example is the discovery of new classes of molecules involved in calcium-regulated nuclear import and nuclear calcium signaling, from the G protein-coupled receptor (GPCR) and myosin families. This review surveys the new state of the calcium and nuclear signaling fields with the aim of identifying the unifying themes that hold out promise in the context of the problems presented by prostate cancer. Genomic perturbations, kinase cascades, developmental pathways, and channels and transporters are covered, with an emphasis on nuclear transport and functions. Special attention is paid to the molecular mechanisms behind prostate cancer progression to the malignant forms and the unfavorable response to anti-androgen treatment. The survey leads to some new hypotheses that connect heretofore disparate results and may present a translational interest.

## 1. Introduction

Despite the recent progress in diagnosis and treatment, prostate cancer remains one of the most common and lethal malignancies [1,2], with approximately 3.3 million men living with the condition only in the United States. The disease is characterized by the comparative ease of the initial diagnosis, a long period of indolence relative to other common cancers, and an abrupt and less predictable progression to the lethal stages. When sharpened up, the questions facing the practitioners and researchers have been formulated as concerning the possibility of treatment for the patients that need it, and the actual need of those for whom treatment is possible [3]. In many cases, the critical events in disease progression from the stage that is believed to merit only watchful waiting to one that is lethal are the emergence of castration resistance and metastasis [4]. Mechanistically grounded prognostic biomarkers and potential drug targets associated with these steps toward poor survival are of particular interest as outcomes of the molecular biological research.

From the perspective of molecular sciences, the puzzle of prostate cancer is encapsulated in a complex perturbation of multiple regulatory pathways and mechanisms. In the face of this complexity, the biological signal integration by the calcium ion (Ca^2+^), in addition to the central importance of gene expression regulation, presents a particular interest. The intracellular propagation of a calcium-mediated signal [5] and the import of downstream molecules into the nucleus [6] can be seen as two common physicochemical events in the dysregulation involving the cell and its environment. Moreover, calcium channels and GPCRs are among the favored “druggable” targets in therapeutics development [7,8,9], and metastasis is manifested primarily by the molecular-motor driven cell motility [10,11]. The unexpected results of the recent experiments implicate the GPCR and myosin molecular motor molecules in the intranuclear calcium signaling and calcium-regulated nuclear transport [12,13]. Motivated by the emergent role of these mechanisms in multiple aspects of prostate cancer cell regulation, below we review the recent advances in molecular biology of prostate cancer with a special focus on calcium signaling and nuclear transport.

## 2. Genomic Background

### 2.1. NKX3.1 Insufficiency

85% of high-grade prostate intraepithelial neoplasia (PIN) and prostate adenocarcinomas display a loss of heterozygosity in the 8p21.2 locus that includes the *NKX3.1* homeobox gene [14]. The incidence correlates with disease grade [15], and there is evidence of an epigenetic downregulation at the locus as well [16]. *NKX3.1* is an early marker of developing prostate epithelium during budding from the urogenital sinus, and its expression throughout the development of the gland is important for ductal branching and expression of secreted proteins [17,18,19]. It is also important in adulthood, when homo- and heterozygous mutants display hyperplasia and often PIN [20,21]. As shown by Lei et al. [22], NKX3.1 stabilizes the tumor suppressor p53, inhibits the activation of protein kinase B (PKB/AKT), and blocks prostate cancer initiation caused by the phosphatase and tensin homolog deleted on chromosome 10 (PTEN) loss in xenografts. At the same time, *NKX3.1* inactivation in mice leads to a deficiency in the response to oxidative stress [23]. In human prostate cell lines, it has been shown that NKX3.1 promotes the response to DNA damage [24] but is ubiquitinated under the action of inflammatory cytokines [25]. Recent experiments have demonstrated, in addition, that the proteolytic control of NKX3.1 expression levels in prostate cancer cells is regulated by mitogens such as epithelial growth factor (EGF) in an intracellular calcium- and protein kinase C (PKC)-sensitive manner [26]. Thus, on the protein level, the effect of most common genomic abnormality that has been identified in prostate cancer converges with the effect of the posttranslational expression regulation by calcium signaling. Additional experiments are needed to establish the relative impact of these two factors at different stages of the disease development.

### 2.2. Amplification and Susceptibility in MYC

The *MYC* oncogene is somatically amplified (as part of the 8q24 region) in a subset of advanced prostate tumors [27,28]. Specifically nuclear MYC was found to be upregulated in many PIN lesions and most carcinomas in the absence of gene amplification [29]. Numerous single nucleotide polymorphisms have been identified in the gene-poor region next to *MYC* that contains *MYC*’s long-range regulatory elements [30,31,32,33]. It has also been established that overexpression of *MYC* in transgenic mice drives formation of PIN as well as progression to invasive adenocarcinoma [34]. The tumors formed under these conditions are characterized by downregulation of *NKX3.1* and a simultaneous upregulation of *Pim1*, *MYC*’s partner in lymphomas. Coexpression of *MYC* and *Pim1*, on the other hand, leads to formation of carcinomas that exhibit the neuroendocrine phenotype [35]. Similarly to the recent results concerning NKX3.1, it has been known for some time that *MYC* in the prostate cancer cells is under the control of calcium-mediated signaling. Specifically, it has been shown that *MYC* is controlled by Notch, which involves the calcium/calmodulin-dependent kinase II (CAMKII)-regulated production of the nuclear import-competent cleaved form of Notch-1 [36]. The crosstalk of this calcium-regulated nuclear signaling pathway, which connects with one of the key regulators of prostate cancer progression, with other factors influencing the intracellular calcium homeostasis appears to be a promising direction for future research.

### 2.3. TMPRSS2-ERG Fusion

Most prostate carcinomas exhibit deletions (less commonly translocations) on chromosome 21q that activate ETS-family transcription factors, usually via an N-terminal fusion with the androgen receptor (AR)-activated gene *TMPRSS2* [37,38,39,40,41]. *TMPRSS2-ERG* is found in approximately 15% of high-grade PINs and 50% of localized prostate cancer [42,43]. In agreement with these clinical sample analyses, AR binding in the lymph node cancer of prostate (LNCaP) cell line brings the *TMPRSS2* and *ERG* loci in physical proximity [44,45]. This finding suggests a role for androgen signaling in the induction of fusions under conditions leading to DNA damage. In this connection, it is interesting that double-stranded breaks also occur under the action of topoisomerase II, which is recruited to the AR-responsive elements by androgen signaling [46]. At the same time, whole-genome chromatin immunoprecipitation analysis has shown that ERG binding can silence AR-responsive genes [47]. Conceivably, this effect may reinforce the physiological hormonal changes in the aging prostate, as well as those accompanying therapeutic castration. Furthermore, in cell culture and transgenic mice, activation of *ETS* expression contributes quantitatively to promotion of invasivity and epithelial-mesenchymal transition (EMT) [38,48,49]. By itself, expression of truncated human *ERG* in mice leads only to a weak PIN [48,49]. It however synergizes with a loss of *PTEN*, leading to a high-grade PIN and carcinoma [50,51]. Collectively, these results put the *TMPRSS2-ERG* fusion at the center of a web of mechanisms responsible for the prostate cancer progression.

Recent work has begun to clarify the relationship of this common genomic condition with perturbations of calcium signaling and nuclear import. Epigenomic profiling has identified the genes that are differentially methylated in the *TMPRSS2-ERG* fusion vs. non-fusion prostate tumors [52]. Among the top-ranked genes is the calcium-channel gene *CACNA1D*, a target of ERG. The most recent extension of this work employed retrospective analysis to demonstrate a negative correlation of the calcium channel blocker use by the prostate cancer patients with both high-grade disease and occurrence of fusion tumors [53]. The latter result can be interpreted as a selective disadvantage of a fusion in the absence of a functional reinforcement by means of the perturbation of calcium signaling. Prostate cancer and benign epithelial prostate cells overexpressing ERG display a characteristic invasivity in vitro [48], an effect that was most recently reversed using peptidomimetic inhibitors of ERG that were fused with a nuclear localization sequence (NLS) peptides to colocalize with ERG in the nucleus [54]. It is remarkable that of the two forms of the fusion protein found in prostate cancer cells, one lacks the NLS and does not enter the nucleus [55]. It binds the fully functional form of the fusion and results in its downregulation on the protein level as well as a reduced expression of MYC. The evidence points to the importance of further study of prostate oncoprotein isoforms that may be characterized by different nucleocytoplasmic partitioning.

The *TMPRSS2-ERG* fusion activity is also associated with Y-box binding protein 1 (YB-1), a genomic-instability response protein that enters the nucleus in response to genotoxic stress [56]. YB-1 contributes to the upregulation of AR during the development of castration resistance [57]. The nuclear localization of this protein has been most recently associated statistically with a poor prognosis for prostate cancer patients [58]. Its accumulation was at the same time shown to be linked to the *TMPRSS2-ERG* fusion, as well as *PTEN* deletion. It appears worth investigating whether the conditions for nuclear import, for example, the calcium-mediated depolarization of the nuclear envelope that is reviewed below, impact the YB-1 regulated downstream effects of the *TMPRSS2-ERG* fusion.

Additionally, the fusion drives expression of Toll-like receptors (TLR), including TLR4 [59]. This results in an increased phosphorylation of nuclear factor κB (NF-κB) on the serine residue that prevents binding of inhibitor of κB (IκB) that masks the NLS. The active fusion isoform and the phosphorylated factor both localize exclusively in the nucleus, as seen also in the orthotopic tumor samples. In vitro, the same experiments demonstrate, the cells’ proliferation is controlled by the transcriptional activity of NF-κB, and the latter is increased with the expression of the fusion product. These findings have led to the speculation that the activation of TLR4 by bacterial lipopolysaccharides, as well as tumor microenvironment proteins, may impact the progression of prostate cancer. Adding to the hypothetical effects that may be induced by the *TMPRSS2-ERG* fusion, it should be noted that NF-κB is known to be able to drive an aberrant ligand-independent nuclear accumulation of AR by direct binding [60]. Conceivably, this may mean that the fusion can aid the cells in bypassing the critical androgen-regulated step during the establishment of androgen independence in the progressing tumor. One more gene activated downstream from *TMPRSS2-ERG* is the chemokine receptor *CXCR4* [61], whose role in intranuclear calcium signaling will be reviewed in Section 8.1.

### 2.4. PTEN Loss

The phosphatase gene *PTEN* undergoes homozygous deletion early in prostate carcinogenesis [62]. The loss has been found to be correlated with the development of aggressive and castration-resistant disease [63,64,65]. At the same time, it has been shown that conditional deletion of *PTEN* in prostate epithelium leads to PIN and adenocarcinoma [66]. Inactivation of *PTEN* in genetically engineered mice was found to cooperate with the loss of function of *NKX3.1* [67], upregulation of *c-MYC* [68], and expression of the human *TMPRSS2-ERG* fusion [50,51]. Among the molecular partners required for tumor formation on the *PTEN* loss background, the studies have identified mammalian target of rapamycin complex 2 (mTORC2) and phosphoinositide 3-kinase (PI3K) isoform p110β [69,70]. It is remarkable that prostate epithelial cells from *NKX3.1*; *PTEN* mutant mice display androgen independence even before development of the cancerous phenotype [71]. If this finding is translatable to the conditions in the human prostate, it indicates sufficiency of the combination of these common early and advanced genomic abnormalities for the critical step in the disease progression. This possibility raises the importance of further investigation, as suggested in Section 2.1, of the calcium-dependent regulation of NKX3.1, which appears to parallel the background genomic effect on the protein level.

In LNCaP cells, PTEN was found to interact with AR directly, resulting in an inhibition of the latter’s nuclear localization and promotion of its degradation [72]. PTEN’s own localization in heterozygous (+/−) prostate cancer cells was found to be predominantly nuclear as assessed by immunofluorescence in vitro [73]. The nuclear retention of PTEN was investigated also on model non-prostate-cancer cell lines [74] and found to be regulated by reactive oxygen species (ROS). The functional importance of the nuclear localization has been demonstrated in the same study in mouse xenograft models. Using human prostate cancer cells expressing mutant forms of PTEN, it was possible to demonstrate that nuclear PTEN acts in concert with p53 to suppress tumor formation. Most recently, the nuclear transport receptor importin 11 has been identified as a tumor suppressor that protects PTEN from proteolytic degradation [75]. This suggest the existence of a secondary function of the molecular interaction that presumably underpins the phosphatase’s nuclear import. Generally, it appears to be an example of the nuclear import serving also as a regulatory mechanism impacting the protein-level expression of a critical tumor suppressor, akin to the chaperone-mediated sequestration mechanisms that will be reviewed in Section 7.

## 3. Developmental Pathways

Gene expression studies suggest that developmental signaling pathways may be reactivated during neoplastic processes in the adult prostate [76,77]. In the mouse, the wingless-integration adenomatous-polyposis-coli (WNT-APC)-β-catenin pathway is seen as contributing to carcinogenesis positively [78,79,80]. Consistent with this is the observation of an enhanced nuclear localization of β-catenin specifically in castration-resistant tumors [81]. In man, on the other hand, the nuclear localization of β-catenin has been seen as negatively correlated with tumor progression [82,83]. Further pointing to an explanation whereby the WNT-β-catenin signaling in prostate cancer development may be stage-specific is the finding that the WNT5A protein is upregulated specifically in the metastatic human samples [84]. The same work also demonstrated the existence of calcium waves that are induced by WNT5A in cultured metastatic prostate cancer cells, as well as this protein’s stimulation of the actin cytoskeleton activity and cell motility (the latter being also under the control of CAMKII). Subsequent experiments showed that the calcium wave induced by this and other WNT proteins results in an intranuclear calcium mobilization and import of β-catenin into the nucleus, which is dependent on a thapsigargin-sensitive depolarization of the nuclear envelope [85]. The results support an interpretation whereby WNT signaling has a dual role as an anti-dedifferentiation regulator and a promoter of cell motility. It will be interesting to determine whether the intranuclear calcium mobilization by WNT impacts the functions of nuclear myosin (reviewed in Section 8.2), and what mechanisms or quantitative properties distinguish it from the one that has for some time been viewed as associated with apoptosis [86,87].

Among other developmental pathways, Hedgehog and fibroblast growth factor (FGF) signaling should be mentioned. Hedgehog signaling plays a significant role in prostate cancer progression [88]. Although experiments on tumor-derived primary cultures were seen initially as pointing toward an autocrine mechanism [89], targeted experimentation identified the mechanism as paracrine signaling to the epithelium from the prostate stroma [90], similarly to other epithelial cancers [91]. Nuclear accumulation of the downstream effector of Hedgehog signaling, transcription factor Gli1, as seen in clinical samples, is associated with metastasis and poor survival [92]. A breaking report [93] identifies the mode of the intracellular Hedgehog signal propagation in the prostate cancer cells as a novel non-canonical pathway involving AR binding-driven nuclear entry of the Gli transcription factors. The contribution of FGF signaling is analogous, in that both epithelial activation of FGF receptor 1 and stromal overexpression of FGF10 play a role [94,95], potentially leading downstream to the activation of extracellular signal-regulated kinase (ERK). Kwabi-Addo et al. [96] call attention to the alternative translation initiation isoforms of FGF2, which display an intranuclear accumulation and are expressed in transgenic adenocarcinoma of the mouse prostate (TRAMP) models, and posit that these isoforms may present a special interest in the context of paracrine signaling, because of the possibility of direct translation of the extracellular signal to the gene expression effects in the target cell. Overall, the ongoing work on the developmental pathways’ dysregulation in prostate cancer emphasizes the need for the elucidation of the pivotal molecular mechanisms behind the calcium-regulated nuclear import, which may involve such novel biophysical principles as gating by the nuclear envelope depolarization.

## 4. Kinase Cascades

The *PTEN* loss of function, which may result, in particular, from the reviewed genomic abnormality, has been shown to cause an upregulation of the AKT/mTOR pathway, primarily via activation of the kinase AKT1 [97,98,99]. An alternative activating mechanism for this signaling pathway is the activating mutation in AKT1 itself, which has been found in human prostate cancer [100]. Experimental expression of the constitutively activated isoform p110β of PI3K is also sufficient for induction of neoplasia in mice [101]. In addition, activation of either AKT or ERK enables androgen-independent growth both in vitro and in vivo [102]. Collectively, these findings establish a broad molecular background among the intracellular signaling kinase classes, whose perturbation may support the development of prostate cancer.

Underscoring the functional importance of the activated kinases’ localization in the cell, it has been shown that tumor suppressor PML recruits both phospho-AKT (pAKT) and its phosphatase PP2 to nuclear bodies [103]. Complicating the picture, loss of *PML* leads to an impairment of the PP2 activity toward pAKT and an accumulation of the latter in the nucleus. The kinase acts in the nucleus by inhibiting FOXO transcription factors, in particular the FOXO3A-mediated transcription of Bim and p27 (Kip1), suggesting a two-pronged mechanism that involves both the suppression of apoptosis and an acceleration of the cell cycle. Promisingly for the translation of this insight into the practice of prostate cancer drug development, the AKT inhibitor ML-9 has recently been found effective at inducing prostate cancer cell death in vitro in a calcium-dependent manner [104].

Adding to the in vitro and in vivo evidence, ERK is frequently found to be activated in the clinical prostate cancer, often at advanced stages and in combination with AKT. As reviewed above, the simultaneous activation promotes progression and androgen independence [102,105]. Moreover, simultaneous targeting of the two pathways was sufficient to inhibit hormone-refractory prostate cancer in the mouse model [106]. Mechanistically, the activation of mTOR and IκB kinase (IKK) by AKT leads to stimulation of NF-κB [107]. Supporting the significance of this regulation, the abnormal NF-κB signaling correlates with androgen sensitivity, metastasis, and outcome in human prostate cancer and the mouse model. The studies implicate both the expression and the nuclear localization of NF-κB [108,109,110,111], and identify among the downstream mechanisms its ability to regulate expression of AR [112].

Phosphorylation of mitogen-activated protein kinases (MAPK) by expression of constitutively active RAF or RAS in the mouse prostate epithelium also leads to a nuclear accumulation of pERK, and this action promotes induction of tumorigenesis [79,113]. However, such mutations are rare in human prostate cancer. Notwithstanding, the collective perturbation of the RAS/RAF pathway via small but collectively significant alterations in the expression of the individual pathway members is prevalent in advanced human prostate cancer [114]. Phospho-MAPK, whether induced by EGF in vitro or prevalent in certain subsets of prostate cancer epithelial cells as assessed by immunofluorescence in vivo, preferentially partitions into the nucleus [115], which can be seen as indicative of the predominantly nuclear downstream functions. These functions remain to be investigated. Taken together, the data reviewed in the last sections demonstrate how a large number of fundamental molecular perturbations behind the development of prostate cancer are interlinked by calcium signaling and the calcium-regulated nuclear import mechanisms (Table 1).

## 5. Anti-Senescence Signaling

A limitless proliferation potential achieved through anti-senescence factors has been proposed to be one of the biological hallmarks of cancer cells [116,117]. The recent characterization of store-operated calcium entry (SOCE) channel components STIM1 and ORAI1 in prostate cancer sheds light on the involvement of calcium in these processes. It was found [118] that the expression of both STIM1 and ORAI1 correlated negatively with the Gleason score (the commonly used histopathological assessment of prostate cancer progression) and was lower in prostate carcinomas relative to the normal tissue. STIM1 was also significantly reduced in hyperplasia. In agreement with these results obtained by immunohistochemistry on patient samples, STIM1 expression was reduced in carcinoma cell lines relative to the benign prostate hyperplasia (BPH) cell line BPH-1, and additionally reduced in the malignant PC3 line relative to the less metastatic DU145. The endogenous expression level differences in these experiments had the expected functional consequences for SOCE activity. Overexpression of the two factors, on the other hand, induced growth inhibition via a cell cycle arrest and senescence, as detected morphologically and by β-galactosidase staining. The apoptosis markers were similarly affected. The complex phenotype was additionally characterized by an increased migration of the cultured cells and a decreased ability of their conditioned medium to induce recruitment of macrophages in vitro. These effects were correlated with a signature of downstream TGF-β signaling activation, manifested in an overexpression of Snail and WNT1 and a rise of p-Smads and nuclear β-catenin, as well as a perturbed secretion of cytokines. On the other hand, inoculation of the STIM1- and ORAI1-overespressing cells into non-obese diabetic, severe combined immunodeficiency (NOD/SCID) mice showed a comparative reduction of tumor growth and a relative loss of BrdU-positive cells and E-cadherin immunostaining. The latter can be seen as indicative of EMT, in agreement with the complex phenotype observed in vitro. An additional complicating finding both in vivo and in vitro has been an induction, in STIM1- and ORAI1-overexpressing cells and tumors, of a relative overexpression of the apoptosis-inhibiting [119,120] form of the tumor necrosis factor (TNF) receptor, decoy receptor 2 (DcR2), even though the anti-apoptotic Bcl-2 and XIAP in these experiments were reduced. A consistent interpretation may be possible, stemming from the fact that DcR2 is, at the same time, an established senescence marker [121,122].

Additional recent findings linking senescence in prostate cancer to calcium signaling pertain to the nuclear factor of activated T-cells c1 (NFATc1), a protein previously implicated in the regulation of expression of prostate specific membrane antigen in an ionomycin-responsive manner in LNCaP cells [123], as well as in the expression of the osteomimicry markers in PC3 and C4-2B cells [124]. NFATc subfamily proteins (the majority of the NFAT types) are activated via dephosphorylation by the calcium/calmodulin-dependent serine/threonine phosphatase calcineurin, upon which the NLS is exposed and the protein undergoes import into the nucleus, where it performs the functions of a transcription activator [125]. In a general agreement with previous work on NFATc1, Manda et al. [126] find the protein expressed in human adenocarcinoma samples (both in the neoplastic epithelium and in the stroma) as well as in tumorigenic prostate cancer cell lines, but not in the epithelium of the normal prostate or benign RWPE-1 cells. Targeted expression of the activated nuclear form of NFATc1 in a mouse model leads to PIN and subsequently adenocarcinoma marked by an elevated expression of proinflammatory cytokines, apparent proliferative influence of NFATc1^+^ cells on the neighboring cells, and castration resistance. Revealingly, MYC was upregulated in both NFATc1^+^ and—apparently via the elevated IL6 and pSTAT3—in the neighboring cells. A double transgenic model with a *PTEN* deletion displayed a synergy of the factors and an accelerated tumorigenesis. Compared with the common *PTEN* deletion model, the prostates were marked by a suppressed expression and nuclear localization of the senescence marker p21 and an insignificant β-galactosidase staining. The mechanism of the p21 suppression may involve the STAT3-SKP2 pathway demonstrated in the gastric and cervical cancer cells [127,128]. Altogether, the data suggest that the calcium-regulated nuclear import step may be critical for the establishment of anti-senescence signaling in advanced prostate cancer.

## 6. Channels and Transporters

### 6.1. Transient Receptor Potential (TRP) Channels

Channels and transporters are among the best-studied molecular classes orchestrating the calcium signaling. In the recent years, considerable attention has been given to the role of TRP channels in the development of prostate cancer. The function of TRPV6, a vanilloid subfamily plasma membrane calcium channel, in particular, is now comparatively well characterized. Earlier termed CaT-like, it is a marker of locally advanced, metastatic, and androgen-insensitive prostate cancer [129]. On mRNA level, in situ hybridization uncovered a correlation of its expression with a number of cancer progression characteristics, including the Gleason score [130]. Remarkably, while its expression is detectable in most androgen-insensitive lesions, the level is reduced relative to untreated tumors. In-vitro studies have shown that this channel, as well as the related TRPC6, increases prostate cancer cell proliferation, acting through NFAT [131,132]. A subsequent detailed immunohistochemical analysis established also the correlation of the TRPV6 expression with prostate-specific antigen (PSA) and TRPC1, and with a lack of the apoptosis marker caspase-3 cleaved fragment [133]. Intriguingly, the presence of TRPV6 in the more advanced tumors, where it is found in luminal cells, was correlated with the appearance of ORAI1 in the same cells, which normally localizes to the basal cells. It has been possible to establish the involvement of TRPV6 in SOCE in prostate cancer cells, as well as its physical association with ORAI1 via STIM1 and TRPC1 under the conditions of SOCE. The latter conditions also induced a translocation of TRPV6 to the plasma membrane, as observed using a confocal microscope. The same study showed that overexpression of TPRV6 in prostate cancer cells in vitro increases their resistance to apoptosis induced, for example, by cisplatin, while in heterotopic xenografts in mice its experimentally manipulated level of expression correlates with the resulting tumor mass. Most recently, an inhibitor of this channel was evaluated in a first-in-human phase I clinical trial with promising results [134]. The work on TRPV6, to date, can serve as a model of calcium-mediated mechanism elucidation leading to translationally relevant results in the prostate cancer field.

Among the other TRP channels, as reviewed most recently by Cui et al. [8], the nonselective cationic channel vanilloid 2 (TRPV2) has been associated specifically with metastatic prostate cancer. Experiments in an earlier TRPV2 study [135] demonstrated the channel’s involvement in the promotion of prostate cancer cells’ migration and invasion both in vitro and in vivo. The effect, accompanied by elevated expression levels of the invasion enzymes MMP2 and MMP9, as well as cathepsin B, appeared to be mediated by an elevated basal cytosolic concentration of Ca^2+^ that was dependent on the channel’s expression level. For progress in system-level understanding of the mechanism behind the emergence of prostate cancer invasivity, it will be of interest to characterize the functional interplay of these effects with the myosin-driven secretion of metalloproteases [136], a process that is likely open to calcium regulation in the light of the molecular dynamics investigations that will be reviewed in the last section.

Another TRP channel, melastatin family 8 (TRPM8, initially termed TRP-P8), was first found to be overexpressed in prostate cancer by Tsavaler et al. [137]. In androgen-responsive prostate cancer cells in vitro, it is expressed on the plasma membrane and in the endoplasmic reticulum (ER), and the level of expression is positively regulated by androgen [138]. The channel is capable of initiating an agonist-induced elevation of the intracellular calcium ion concentration. In keeping with the general theme of fine-tuned homeostasis, which is widely accepted to characterize the calcium regulation [139], both the agonist and antagonist action on TRPM8 in in-vitro experiments was able to induce apoptosis. In the same experiments, the channel was found to be expressed at a low and unregulated level in androgen-insensitive cells. This finding agrees with the loss of TRPM8 in transition to androgen independence that was detected in a genome-wide expression profiling study [140]. Subsequent work [141] found that experimental re-expression of this channel in androgen-insensitive cells reduced their migration in vitro. At the same time, in non-prostate-cancer model cell culture, PSA was able to potentiate the TRPM8 agonist-induced calcium current by enhancing trafficking of the channel to the plasma membrane via its phosphorylation downstream of the bradykinin 2 receptor signaling pathway. In resection samples and primary cultures, the plasma membrane localization and activity of this channel was found to be characteristic of the luminal cells of normal tissue, BPH, and especially in situ tumors, whereas the dedifferentiated transit amplifying and intermediate phenotype retained primarily the endoplasmic isoform [142]. As pointed out by the authors of this investigation, one functional consequence of the endoplasmic localization of the channel may be the apoptosis-resistant state characterized by a lowered intraendoplasmic calcium concentration, which had been seen in other studies of advanced prostate cancer [143,144]. Thus, the channel’s role appears to be different, depending on the stage of prostate cancer, and change from its expression maintaining the pro-survival calcium homeostasis to its partial loss potentially contributing to the development of metastatic potential in parallel with the acquisition of androgen independence.

A recent study [145] has implicated a second melastatin family TRP channel, TRPM7, in the positive regulation of prostate cancer cells’ migration, invasion, and expression of MMPs, as well as in downregulation of E-cadherin that accompanies EMT. Furthermore, the related TRPM4 has a function in the proliferation of prostate cancer cells, according to a breaking report [146]. In this instance, the downstream cascade includes a relative dephosphorylation of β-catenin and an elevation of this regulator’s nuclear localization. Completing the present picture of a broad and mechanistically diverse involvement of the melastatin subfamily channels in the prostate cancer development, TRPM2 is one more known member that controls proliferation of prostate cancer cells by inhibiting the nuclear ADP-ribosylation [147]. The intracellular localization of TRPM2, which is associated with the plasmalemma of benign prostate epithelial cells, is altered in tumorigenic prostate cell lines, where it appears in intranuclear clusters. It will be interesting to investigate if the channels of this broad class are similar to the GPCRs to be reviewed in Section 8.1 in exhibiting nuclear translocation and influencing the trans-nuclear envelope potential and intranuclear calcium concentration in prostate cancer cells. Their downstream targets, hypothetically, could include the physical nuclear organization and transcription-related functions of nuclear myosins (see Section 8.2).

### 6.2. Other Channels and Transporters

The other types of channels and transporters that have been implicated in the development of prostate cancer include molecules responsible for a remarkably diverse set of calcium homeostasis mechanisms, such as voltage-gated and calcium-activated channels, as well as intracellular pumps. In particular, there is pharmacological evidence of a contribution from T-type calcium channels, including, specifically, Ca_v_3.2, to the proliferative activity of prostate cancer cells in vitro and to cancer progression in xenograft experiments [148,149]. A similar involvement of this type of channels has been documented in other tumor types [150]. The Ca_v_3.2 isoform may also be involved in neuroendocrine differentiation in prostate cancer, which is associated with castration resistance, distant metastasis, and poor prognosis [151]. Revealingly, induction of neuroendocrine differentiation of LNCaP epithelial prostate cancer cells by androgen deprivation in vitro was found to be accompanied by an upregulation of Ca_v_3.2 and an increase in the associated calcium current [152,153]. These results suggest that a calcium-mediated reinforcement loop similar to the one already discussed in connection with the *CACNA1D* gene (which encodes Ca_v_1.3, see Section 2.3) may be contributing to the neuroendocrine differentiation in the androgen-deprived prostate.

An intriguing connection to the cytoskeleton signaling has recently been uncovered that involves a plasma membrane channel that is calcium-activated but conducts potassium ions. The big potassium calcium-sensitive (BKCa) channels are expressed in excitable cells [154] and aberrantly in cancers of various origin [155]. They are overexpressed in a significant subset of advanced prostate cancers, and inhibiting their expression can suppress PC3 cell proliferation in vitro [156]. A recent study [157] has extended these results, using a xenograft model, and additionally demonstrated a stimulating effect of these channels’ overexpression on cell migration and invasion. Interestingly, the effects depended only slightly on the channels’ conductance, but were exerted instead in an integrin- and phosphorylated focal adhesion kinase (FAK)-dependent manner. The immunofluorescence colocalization and immunoprecipitation showed a formation of a BKCa-αVβ3 integrin complex, accompanied by recruitment and phosphorylation of FAK. The possible interplay of this mechanism with the tumor stroma’s influence on the focal adhesion signaling, as well as the potential feedback from the extracellular matrix modification by the invasive epithelial cells themselves, present interest for future research.

The sarcoendoplasmic calcium pump SERCA’s expression in epithelial prostate cancer cells is upregulated by the action of EGF, dihydrotestosterone (DHT), or serum, according to the in-vitro studies on LNCaP cells [158]. Stimulation of proliferation caused by these agents is suppressed by the SERCA inhibitor thapsigargin. Thus, the pump appears to be both an intermediary of the response to mitogens and subject to the downstream regulation caused by these factors, which may sensitize the tumor to the subsequent stimulation. Although the SERCA isoform implicated by the cited study was the non-tissue specific isoform (2B), a prostate-selective thapsigargin analog has been designed [159] and, most recently, reached phase II in the clinical trials for prostate cancer [8].

In addition to the reviewed roles of calcium-release activated calcium channel protein ORAI1 in senescence and SOCE, the channel appears to be involved also in the capacitative entry mediating apoptosis in androgen-sensitive prostate cancer cells in culture [160]. The androgen dependence of expression of ORAI1 itself, which was demonstrated in this study, raises the possibility of its downregulation being part of the mechanism of the emergence of aggressive cancer following the androgen deprivation therapy. Extending these results, the store-independent entry was recently associated with prostate cancer-specific enhanced expression of ORAI3 and formation of a heteromeric channel with ORAI1 [161]. Interestingly, the channel is gated by arachidonic acid, opening a new link between calcium signaling and the regulation by metabolism and inflammation.

The mitochondrial calcium uniporter, which contributes to the proapoptotic accumulation of calcium in mitochondria, is a comparatively new pump that has been studied in relation to cancer [8]. In-silico screens pointed to a negative regulation of its expression by miR-25, and this prediction was borne out by the experimental measurements [162]. Established prostate cancer cell lines express large quantities of this micro-RNA and are, at the same time, characterized by suppressed levels of the uniporter. Experimental restoration of its expression has been shown to sensitize prostate cancer cells in vitro to apoptosis. This is a promising direction for a further investigation in vivo*.*

An important apoptotic pathway is mediated by a sustained Ca^2+^ flux from the ER to mitochondria through the endoplasmic inositol 1,4,5-trisphosphate receptors, InsP3R [163]. The regulation of this mechanism has been linked to the deficiency of the *PTEN* gene, which, as reviewed above, is common in prostate cancer. The role of InsP3R has been elucidated in a series of experiments on established prostate cancer cell lines, which similarly exhibit the *PTEN* inactivation [164]. LNCaP cells, in particular, are characterized by a frameshift mutation in *PTEN*, and PKB/ACT in these cells was found to be constitutively phosphorylated and activated in the PI3K-dependent manner [165]. The phosphorylation in these experiments has proved determinative of the cells’ resistance to apoptosis upon withdrawal of the serum factors. Subsequent work [166] has traced the mechanism of the resistance to phosphorylation of InsP3R by AKT. Most recently, a similar effect has been described in PC3 cells, where it is mediated by the lack of competition from the deficient *PTEN* product for binding to InsP3R3, which opens the latter to binding the F-box protein FBXL2 [167]. FBXL2 has the function of the receptor subunit of the ubiquitin ligase complexes, and can target InsP3R3 for degradation. The different mechanisms in the lymph node- and bone-metastatic cell lines may be indicative of diverging functions that can be discovered even for the comparatively well-characterized molecular classes at the different stages of prostate cancer progression. While the conditions-dependent multifunctionality may play the role of a complicating factor in drug development, it can also present an opportunity to more selectively target the tumor development at its specific crucial steps.

Finally, in the discussion of the channels involved in calcium signaling in prostate cancer, ryanodine receptors (RyR) have also been found to be expressed in prostate cancer cell lines [168]. The isoforms RyR1-3 vary in quantity depending on the cell line, a finding that should be further investigated, as it may be indicative of modification of the response mediated by these channels, depending on the stage in the tumor development or its environment. Calcium release is observed under the action of the RyR agonist caffeine and has been found to mildly promote apoptosis of the lymph-node metastatic LNCaP cells in vitro [169]. A synthetic agonist 4-chloro-*m*-cresol was also effective at eliciting this calcium response in these experiments, pointing to a possibility of targeting the receptor through a prostate-selective drug design in the future. On the whole, the calcium and calcium-operated channels and transporters remain one of the most systematically studied molecular classes in relation to their roles in prostate cancer (Table 2).

## 7. Additional Topics

### 7.1. Calpain Proteolysis

Upon elevation of the intracellular calcium concentration in prostate cancer cells, as demonstrated in experiments in vitro [170], the calcium-regulated protease calpain cleaves β-catenin, producing a stable, transcriptionally active fragment that enters the nucleus. At the same time, microarray experiments demonstrated an elevation of the calpain expression in metastatic prostate cancer, which may suggest sensitization of this nuclear import pathway to calcium signaling in cells undergoing the transition to malignancy. Alongside the calcineurin-NFAT (Section 5) and calmodulin-myosin (Section 8.2) mechanisms, the calpain-β-catenin mechanism can be seen as part of an emerging paradigmatic triad representative of the mechanistically diverse calcium-regulated nuclear import pathways, many of which probably remain to be elucidated.

Another target for the calcium-induced cleavage by calpain in prostate cancer cells is E-cadherin, whose inactivation is characteristic of adenocarcinomas and is often seen as a marker for EMT [171]. The intracellular calcium-induced calpain proteolysis of FAK [172], at the same time, is a contributing factor to the modulation of cell adhesion in developing prostate cancer. The likely BKCa-mediated crosstalk with the mechanism reviewed in the last section is a promising avenue for future research. On the whole, the data on calpain demonstrate a remarkably multifaceted role of the calcium-regulated proteolysis in prostate cancer development and emphasize the simultaneous challenge and opportunity for the systems-based design of novel therapeutics that could reverse the dysregulation of this process.

### 7.2. 14-3-3 Mediated Nucleocytoplasmic Redistribution

As reviewed above (Section 4), AKT is one of the key signaling kinases involved in prostate cancer development, which exerts its effects, in particular, by inhibiting the FOXO3A transcription activator of the proapoptotic factor Bim through a mechanism that involves nucleocytoplasmic partitioning. Additional light has been shed on this issue by the work that has implicated the chaperone protein 14-3-3 [173]. The serine phosphorylation of FOXO3A by AKT creates a binding site for 14-3-3 and leads to retention of the complex in the cytoplasm, sequestering the transcription factor. The cytoplasmic localization of FOXO3A and 14-3-3 is correlated with the Gleason score in human samples and with disease progression in TRAMP mice [174]. In the reviewed work by Trotman et al. [103], it was shown, in particular, that pAKT in the mouse model prostate lesions accumulates in the nuclei. The mechanistic significance of this fact has been illuminated by the results that were obtained on non-prostate-cancer cells in vitro [175]. In the latter experiments, the nuclear pAKT was found to phosphorylate FOXO3A/FKHL1 immediately prior to the 14-3-3 binding and nuclear export of this transcription factor. The chaperone-mediated sequestration and nuclear export induced by the intranuclear activated kinase is an intriguing element of the nuclear transport-related mechanistic repertoire of prostate cancer, which merits further study.

### 7.3. Autonomic-System Regulation

In addition to the *PTEN* inactivation and constitutive activation of AKT, an acetylcholine-induced and calcium-dependent pathway leading to this kinase’s activation has been uncovered recently [176]. Following the in-vivo indications that the autonomic, including cholinergic, nerve infiltration promoted prostate cancer development [177], the in-vitro work by Wang et al. [176] revealed the existence of autocrine acetylcholine signaling in epithelial prostate cancer cells, which proceeds through the muscarinic GPCR CHRM3 and a Ca^2+^ influx. Phosphorylation of AKT under these conditions was found to be mediated by calmodulin and CAMK kinase (CAMKK). CHRM3 is known to be upregulated in human prostate cancer samples, and experiments in vivo and in vitro have implicated the autocrine mechanism in the prostate cancer cells’ proliferative and migratory potential. An antagonist treatment in vivo also increased the percentage of apoptotic cells and reduced the castration-resistant tumor growth. The most recent extension of this work has implicated the autocrine calcium-dependent mechanism in BPH and maintaining the prostate epithelial progenitor cells in the proliferative state [178]. It has also been demonstrated in LNCaP cells that carbachol-induced CAMKK-dependent blockage of apoptosis is mediated by AKT phosphorylation of the Bcl-2 associated death promoter protein BAD [179]. The effect on BAD is linked to its 14-3-3 binding and sequestration in the cytosol from the mitochondria, as shown in non-prostate-cancer cells [180]. Similar to the cytoplasmic sequestration of FOXO3A from the nucleus (last section), this mechanism adds complexity to the web of the spatially-restricted interactions of the molecules involved in the intracellular transport downstream of calcium signaling.

It is interesting to note that although the sympathetic, adrenergic effects in the Magnon et al. experiments [177] were found to be mediated by the stromal β1 and β2 receptors, a similar adrenergic effect on proliferation of primary prostate epithelial cells was demonstrated in the reviewed work of Thebault et al. [131], where it was mediated by α1 receptors. The downstream adrenergic signaling seen in the epithelial cell culture proceeded through a store-independent calcium entry via the TRPC6 channels, calcineurin, and NFAT-dependent transcription, thereby implicating the reviewed (Section 5) calcium-dependent nuclear import pathway.

## 8. New Molecular Classes in Calcium Signaling to the Nucleus

### 8.1. GPCRs

Alongside the adrenergic receptors, whose role was reviewed above, the chemokine cysteine (C)-X-C receptor 4 (CXCR4) is another GPCR that is remarkable for its involvement in the nuclear calcium signaling. It has been implicated in bone tropism of prostate cancer cells [181], in which it participates through its interaction with its bone-associated agonist stromal cell-derived factor 1 (SDF-1). It has also been found to promote migration and invasion of prostate cancer cells under the conditions when PTEN is inactivated by ROS [182]. It resides on the plasma membrane but also localizes specifically to the nuclei in human prostate cancer samples, as revealed by means of immunohistochemistry [13]. It similarly accumulates in the nuclear fractions of cultured prostate cancer cells, possesses an NLS, and associates with the nuclear import receptor transportin-β1. Exposure to SDF-1 results in dissociation of the α subunit from the CXCR4 in the nuclear fraction, leading to intranuclear calcium release. In addition to the identification of a new class of nuclear calcium signaling molecules associated with prostate cancer, this recent study points to a novel mechanism of escape from any therapeutic receptor antagonist action that is limited to the cell surface.

### 8.2. Myosins

Non-muscle myosins have recently been shown to have multiple functions in prostate cancer. The longest-known functions of the myosin family concern cell motility, and recent work on prostate cancer cells has finely delineated this class of functions among the myosin family members including IB, IXB, X, and XVIIIA [183]. The contributions of these molecules to the shaping of the actin cytoskeleton and motile morphology of prostate cancer cells underpin mechanically the acquisition of a metastatic phenotype by the originally non-motile epithelial cells [184]. Myosin IC too has long been known as a contributor to cell migration and to intracellular transport in various cell types [185]. The most recent results have implicated it in the prostate cancer cell migration and invasion [136]. The three isoforms of this protein, including the recently discovered one (A) that is associated with metastatic prostate cancer [186,187]; and the one (B) originally described as the nuclear myosin [188], differ kinetically, according to the latest work [189].

Following the discovery of the NLS [190] within the IQ domain of myosin IC that mediates the interaction with apo-calmodulin, it was demonstrated that in prostate cancer cells, elevation of the intracellular calcium concentration drives the nuclear import of this myosin [12]. This import depends on importin-β1 and is presumed to be mediated by the unmasking of the NLS when, as established for myosin IC in vitro [191], the calcium-calmodulin dissociates from the IQ domain. The most recent work [192] has demonstrated that binding to phosphoinositides, mediated in part by the NLS region, contributes to the nuclear import of myosin IC. It will be of interest to determine whether similar mechanisms regulate the nucleocytoplasmic partitioning of the other myosins known to localize to the nucleus, such as subfamilies II, V, VI, X, XVI, and XVIII [193].

The nuclear functions have to date been best characterized for myosin IC isoform B. They include promotion of transcription, which is effected through an association with RNA polymerases I and II [194,195,196,197,198,199,200]. This myosin isoform also associates with RNA transcripts and pre-ribosomal units, participating in the export of the ribosome subunits from the nucleus [201,202]. The movements of chromosomes inside the nucleus similarly depend on nuclear myosin and actin [203,204]. Myosin IC isoform C—long thought to be cytoplasmic but now recognized as the most responsive to the calcium-regulated nuclear import in prostate cancer cells [12]—displays a capacity for interaction with RNA polymerase II and for maintaining the level of polymerase I activity [205]. Since calmodulin, acting as the light chain, modulates the motor properties of myosin IC [206] and itself undergoes nuclear import via the facilitated pathway [207], it appears promising to investigate next the intranuclear calcium regulation of the myosin functions in prostate cancer.

The tumor-suppressor qualities of myosin IC have recently been characterized in non-prostate-cancer cells [208]. Partially similar results involving suppression of anchorage-independent growth had previously been obtained in relation to myosin XVIII in other cancers, as reviewed by Oudekirk and Krendel [10], and most recently in a clinical association study for myosin VC in colorectal cancer [209]. At the same time, in breast and prostate cancer *MYO18A* (which encodes the non-muscle myosin XVIIIA) appeared to be an oncogene [210]. Future work should be able to shed light on the possible reciprocal regulation by means of the calcium-controlled switch between the cytoplasmic and nuclear functions of the non-muscle myosins, leading to a unified picture of these phenomena.

## 9. Summary and Outlook

The intracellular calcium signal and the nuclear entry of the activated factors can be seen as dual, linked integrating events that undergird the molecular complexity behind the critical stages of prostate cancer development (Table 1, Table 2 and Table 3). As these multiple examples in the reviewed literature from the recent years show, the nuclear vs. cytoplasmic localization frequently presents a unique differentiating feature that is associated with a prognosis for progression of the disease. Although localized calcium signals have the capacity to channel the information transmitted by the specific pathways, the influences impinging on the intracellular calcium homeostasis establish a network for a physical integration of the conditions of the tumor microenvironment. Of special interest from the molecular biological viewpoint are the events linking the calcium signaling to the regulation of the nuclear entry, as well as the dynamic steady-state nucleocytoplasmic distribution of the transcription regulators and cytoskeletal effector molecules. The polyfunctionality and novel intracellular localizations of the previously known factors in prostate cancer development that have been uncovered in the most recent work point, on the one hand, at a greater complexity that lies ahead in the molecular exploration of the disease mechanisms. On the other hand, however, they hold out a promise for a mechanistically grounded validation of new prognostic markers and potential targets for drug development, which would be able to disrupt the critical nodes in the prostate cancer network.

## Figures and Tables

**Table 1:** General regulatory factors involved in the calcium and nuclear signaling in prostate cancer. The molecular factors and their cell-physiological effects are categorized, and the corresponding calcium and nuclear events sketched out in the approximate order of their treatment in the text.

Category	Factor	Physiology	Partners	Calcium Regulation/Nuclear Signal
genomic	*NKX3.1* insufficiency	development	p53, AKT, PTEN	Ca^2+^-regulated proteolysis
	*MYC* amplification	cell cycle	NKX3.1, PIM1	Ca^2+^-regulated Notch nuclear entry
	*TMPRSS2-ERG* fusion	EMT	PTEN, *CACNA1D*	NF-κB nuclear entry, Ca^2+^ channel expression
	*PTEN* loss	hormone independence	mTORC2, PI3K	AR nuclear localization
developmental	WNT	cell motility	APC	Ca^2+^ wave, β-catenin nuclear entry
	Hedgehog	signaling from stroma	AR	Gli1 nuclear entry
	FGF	signaling from stroma	ERK	nuclear entry of alternative translation isoforms
kinases and phosphatases	AKT-mTOR	apoptosis	FOXO3A, Bim, p27	pAKT accumulation in nuclear bodies, Ca^2+^-dependent inhibition
	ERK, MAPK	hormone independence	RAS, RAF, AKT	pERK/pMAPK accumulation in nucleus
	calcineurin	anti-senescence	NFATc1, MYC, IL6, STAT3	NFATc1 nuclear import

**Table 2:** Calcium and nuclear signaling in prostate cancer that is associated with specific channels and transporters.

Category	Channel/Transporter	Related Cell Physiology	Partners	Calcium Regulation/Nuclear Signal
TRP channels	TRPM2	proliferation	nuclear ADP-ribosylase	channel accumulation in nuclear clusters
	TRPM4	proliferation	β-catenin	β-catenin nuclear accumulation
	TRPM7	EMT	E-cadherin, MMPs	PM Ca^2+^ channel
	TRPM8	apoptosis	AR, PSA	lowered ER Ca^2+^
	TRPV2	invasion	MMPs	elevated basal Ca^2+^
	TRPV6	proliferation	NFAT, ORAI1, STIM1	SOCE
other channels, transporters	BKCa	invasion	integrin	Ca^2+^-activated phosphorylation of FAK
	SERCA	proliferation	EGF, DHT	ER Ca^2+^
	ORAI1	apoptosis	ORAI3, arachidonic acid	capacitative entry via the gated heteromer
	mitochondrial uniporter	apoptosis	miR-25	mitochondrial Ca^2+^
	InsP3R	apoptosis	AKT, PTEN, FBXL2	ER-to-mitochondria current

**Table 3:** Additional molecular classes involved in the calcium and nuclear signaling in prostate cancer.

Category	Factor	Physiology	Partners	Calcium Regulation/Nuclear Signal
proteases	calpain	metastasis, EMT	AR, β-catenin, E-cadherin	nuclear entry of cleaved β-catenin
chaperones	14-3-3	apoptosis	AKT, FOXO3A, Bim	nuclear export of FOXO3A
autonomic regulation	CHRM3	castration resistance	CAMKK, AKT, BAD	acetylcholine-induced Ca^2+^ influx
	adrenergic receptors	proliferation	TPRC6, calcineurin, NFAT	store-independent entry
GPCRs	CXCR4	bone tropism	SDF-1, PTEN, ROS, importin-β1	nuclear entry, intranuclear Ca^2+^ release
molecular motors	myosin IC	migration, invasion, metastasis	calmodulin, importin-β1, RNA polymerase I and II, nuclear actin	Ca^2+^-induced nuclear entry

## Abbreviations

AR	Androgen receptor
BKCa	Big potassium calcium-sensitive (channel)
BPH	Benign prostate hyperplasia
CAMK	Calcium/calmodulin-dependent kinase
CAMKK	Calcium/calmodulin-dependent kinase kinase
DcR2	Decoy receptor 2
DHT	Dihydrotestosterone
EGF	Epithelial growth factor
EMT	Epithelial-mesenchymal transition
ER	Endoplasmic reticulum
ERK	Extracellular signal-regulated kinase
FAK	Focal adhesion kinase
FGF	Fibroblast growth factor
GPCR	G protein-coupled receptor
IκB	Inhibitor of κB
IKK	Inhibitor of κB kinase
InsP3R	Inositol 1,4,5-trisphosphate receptor
LNCaP	Lymph node cancer of prostate (cell line)
MAPK	Mitogen-activated protein kinase
mTORC2	Mammalian target of rapamycin complex 2
NFAT	Nuclear factor of activated T-cells
NF-κB	Nuclear factor κB
NLS	Nuclear localization signal (sequence)
PI3K	Phosphoinositide 3-kinase
PIN	Prostate intraepithelial neoplasia
PKB	Protein kinase B
PKC	Protein kinase C
PSA	Prostate-specific antigen
PTEN	Phosphatase and tensin homolog deleted on chromosome 10
ROS	Reactive oxygen species
RyR	Ryanodine receptor
SDF-1	Stromal cell-derived factor 1
SOCE	Store-operated calcium entry
TLR	Toll-like receptor
TRAMP	Transgenic adenocarcinoma of the mouse prostate
TRP	Transient receptor potential (channel)
YB-1	Y-box binding protein 1
